# Effects of Tai Chi versus Proprioception Exercise Program on Neuromuscular Function of the Ankle in Elderly People: A Randomized Controlled Trial

**DOI:** 10.1155/2012/265486

**Published:** 2012-12-24

**Authors:** Jing Liu, Xue-Qiang Wang, Jie-Jiao Zheng, Yu-Jian Pan, Ying-Hui Hua, Shang-Min Zhao, Li-Yan Shen, Shuai Fan, Jiu-Gen Zhong

**Affiliations:** ^1^College of Chinese Wushu, Shanghai University of Sport, Yangpu, Shanghai 200438, China; ^2^Department of Sport Rehabilitation, Shanghai University of Sport, Yangpu, Shanghai 200438, China; ^3^Department of Rehabilitation Medicine, Huadong Hospital, Fudan University, Shanghai 200040, China; ^4^Department of Sports Medicine, Huashan Hospital, Fudan University, Shanghai 200040, China

## Abstract

*Background*. Tai Chi is a traditional Chinese medicine exercise used for improving neuromuscular function. This study aimed to investigate the effects of Tai Chi versus proprioception exercise program on neuromuscular function of the ankle in elderly people. *Methods*. Sixty elderly subjects were randomly allocated into three groups of 20 subjects per group. For 16 consecutive weeks, subjects participated in Tai Chi, proprioception exercise, or no structured exercise. Primary outcome measures included joint position sense and muscle strength of ankle. Subjects completed a satisfaction questionnaire upon study completion in Tai Chi and proprioception groups. *Results*. (1) Both Tai Chi group and proprioception exercise group were significantly better than control group in joint position sense of ankle, and there were no significant differences in joint position sense of ankle between TC group and PE group. (2) There were no significant differences in muscle strength of ankle among groups. (3) Subjects expressed more satisfaction with Tai Chi than with proprioception exercise program. *Conclusions*. None of the outcome measures on neuromuscular function at the ankle showed significant change posttraining in the two structured exercise groups. However, the subjects expressed more interest in and satisfaction with Tai Chi than proprioception exercise.

## 1. Introduction

An increasingly aging population presents a global challenge to human society. Such a population shift arises from two demographic effects: increasing longevity and declining fertility [1, 2]. Based on World Health Organization projections, the proportion of the global population that is 60+ years old is expected to increase from 10.0% in 2000 to 21.8% in 2050, and then to rise to 32.2% in 2100 [3]. Due to decreased fertility, China and many other developing countries are going through more rapid fertility transitions than these projections predict. These countries will experience even faster population aging in future years than currently developed countries [3, 4].

Impaired motor performance in elderly people is often characterized by a slowing of movement, a decrease in muscle strength, and a loss of fine motor coordination [5]. These impairments can increase the likelihood of fall in elderly people, as well as decrease these individuals' ability to participate in standard activities of daily living [6, 7]. Thus, realizing an effective method of improving the neuromuscular function of elderly people could be expected to improve quality of life and to reduce social medical costs [8].

Physical activity is an effective strategy for improving neuromuscular function, particularly among the elderly population [5, 8–10]. However, many forms of physical activity are either too intense or too monotonous for older adults to maintain over an extended period of time. 

Tai Chi (TC) is a popular form of exercise among older adults, especially in Asia. This activity involves a series of slow, smooth, and graceful movements, with an emphasis on smooth coordination of the eyes, head, body, and upper and lower extremities; for these reasons, TC is assigned special significance in the daily routine in many older adults [11]. Elderly TC learners may chat and learn from each other as they practice these skills, which can increase learning motivation and maintain a steady exercise habit. In addition, TC learners can practice by themselves at any time and in any place. An added benefit is that TC programs do not require any special equipment, which further adds to this activity's convenience and accessibility.

Numerous studies have investigated TC as an intervention for a wide variety of health problems, especially balance and musculoskeletal disease [12–14]. Li and his colleagues conduct a rigorous, multicenter randomized controlled trial with a large sample size in the New England Journal of Medicine, and they found that TC was more effective in improving postural stability in limits-of-stability tasks than a resistance-based exercise or low-impact stretching [15]. However, it is not yet known whether a proprioception exercise (PE) program has better long-term effects on the neuromuscular function of the ankle than a TC program. Ankle function is of special concern to older adults, as failure of this joint can lead to balance problems and increased risk of fall. Hence, we designed the present study to examine the effects of a 16-week TC training program versus 16 weeks of PE on the proprioceptive function and muscle strength of the ankle in elderly subjects. Here, we hypothesized that a TC program would lead to greater improvements in proprioceptive function and muscle strength outcomes than an alternative PE program.

## 2. Methods

### 2.1. Subjects

Subjects (*n* = 60) were recruited from several community elderly centers in Shanghai, China. Subjects were required to have had no previous experience in TC, as well as no regular physical exercise habits. A computer-generated random-number sequence randomly assigned all subjects to either a TC (*n* = 20; mean age ± SD, 70.5 ± 2.1 y), PE (*n* = 20; mean age ± SD, 72.8 ± 2.3 y), or control group (*n* = 20; mean age ± SD, 68.6 ± 1.6 y). The TC group and the PE group both performed the assigned exercise twice a week (45 min/session) for 16 weeks. The control group had no regular physical exercise habits.

Inclusion criteria are the following: (1) be aged 60–85 years old; (2) score at least 24 on the Mini-Mental State Examination (MMSE) to show that they had no cognitive impairment [16], using the Chinese version validated by Chiu et al. (1994) [17]; (3) demonstrate a sufficient active range of motion in their upper limbs to perform the requisite finger-pointing tasks, which required subjects to flex and extend their shoulder, elbow, wrist, and fingers; (4) demonstrate through the Independent Activity of Daily Living test that they could be considered independent in activities of daily living [18].

Exclusion criteria are the following: (1) cardiovascular pathologies such as symptomatic cardiovascular disease or uncontrolled hypertension; (2) previous experience in TC; (3) any musculoskeletal disease referred to the lower limbs such as low back pain, serious arthritis, and ankylosing spondylitis; (4) any pathology affecting lower extremity function such as stroke, Parkinson's disease, or any other disabling neurologic illness.

### 2.2. Procedure

Prior to initiation of the study, all subjects completed a questionnaire that asked for such details as the subjects' past and present job status and their medical history. They also completed the MMSE and the Activity of Daily Living test and described their exercise habits (frequency and time/session). TC and PE subjects were asked to exercise twice a week (45 min/session) for 16 weeks at the Shanghai University of Sport. Joint position matching and muscle strength tests were conducted at baseline and after the 16-week intervention. Subjects completed a satisfaction survey after completing the 16-week intervention.

### 2.3. Testing Protocol

The assessment test was divided into two sections. The first section assessed subjects' joint position matching ability of the ankle in different degrees. Then, after 30 min of relaxation, the strength of the subjects' ankle dorsiflexors and plantar flexors was evaluated. All tests were performed on both legs of each subject. 

Subjects performed a submaximal warm-up exercise (50–60 W) on a bicycle ergometer (MOTOmed viva2, Reck, Germany) for 5 min prior to the muscle tests. A Biodex System 3 isokinetic dynamometer (Biodex Medical Systems, Shirley, New York, USA) was used to measure peak torque, peak torque/weight, and ankle joint position sense (JPS).

#### 2.3.1. Ankle Joint Position Passive Matching Test [19]

Each subject was positioned semirecumbent on the associated special testing chair, with the calf of the tested leg resting on a 40 cm high platform. The hip and knee were positioned at a 45° flexion, and the talocrural joint was in neutral position. The bare foot of the subject was aligned with the axis of the dynamometer and attached to the footplate by a very small wrap to reduce cutaneous receptor input. During testing, subjects kept their eyes closed and wore headphones with music playing to eliminate visual and auditory stimuli from the testing apparatus. There were two reference degrees: (1) ankle at 10° inversion and (2) ankle at 20° inversion. 

The subject's foot was first passively moved by the investigator to the maximal inversion or eversion position. The investigator then moved the foot to the two reference positions. This test position was maintained for 10 s, with each subject instructed to concentrate on the position of the foot. The foot was then passively brought to maximal inversion or eversion and moved passively back toward eversion or inversion with a speed of 1°/s. The subject was instructed to push on a stop button when he or she thought that the test position had been reached. This trial was repeated three times, and the error with which the subject reproduced the initial position was subsequently calculated. The three absolute error values were averaged, and the average value was termed the absolute angle error.

#### 2.3.2. Muscle Strength Test [20]

We used a Biodex System 3 dynamometer to determine isokinetic peak torque and peak torque/weight values for reciprocal concentric plantar flexion to dorsiflexion movements of the ankle. Subjects were tested in a semi-recumbent position with 30° of seat-back tilt. The ankle was in 10° plantar flexion. The knee of the tested ankle was in extension to minimize substitution from the hamstrings and other tibial rotators. Dynamometer and chair adjustments were made to align the midline of the foot with the midline of the patella. Two straps were wrapped around the extremity proximal to the patella and the pelvis to minimize movements of the hip and knee during testing. Isokinetic contractions were performed at an angular velocity of 30°/s. 

Prior to testing, each subject performed a warm-up exercise of three submaximal repetitions to familiarize themselves with the equipment. For the isokinetic test, the subjects were instructed to push the foot away from them and pull it toward them at maximum velocity through the full available range of motion for each repetition. Peak torque was determined as the highest torque generated from the three trials. Peak torque/body weight is an important consideration for improved comparison among subjects of varied body types. None of the subjects noted any discomfort while testing.

### 2.4. Training Program

#### 2.4.1. The TC Intervention

Subjects participated in 45 min TC sessions twice weekly for 16 weeks. Classes were taught by a Tai Chi master with more than 10 years of teaching experience. In the first session, we explained TC theory and procedures and provided subjects with printed teaching materials, including TC principles, practicing techniques, and safety precautions for the elderly. For the remaining sessions, each subject practiced TC under the instruction of the Tai Chi master. Each session included (1) 5 min of warm-up and a review of TC principles, (2) 30 min of TC movement, (3) 5 min of breathing techniques, and (4) 5 min of cooldown. The program consisted of 24 forms from classic Yang-style Tai Chi, with minor modifications that were suitable for older adults. We encouraged subjects to participate in their usual sports activities, but to not engage in extra strength training.

#### 2.4.2. The Proprioceptive Exercise Intervention

Proprioceptive exercise classes were led by a registered physical therapist for 45 min, twice per week, over 16 weeks. The exercise protocol emphasized static and dynamic balance exercises, including transitions between differing sensory conditions and functional everyday movements. Each lesson incorporated a similar general plan as follows: (1) 5 min warm-up; (2) 20 min of static balance exercises such as squats (two-leg stance) and one-leg stance; (3) 15 min of dynamic balance exercises such as jogging, sideways walking or running with crossovers, forward walking or running in a zigzag line, or backward walking or running in a zigzag line; (4) 5 min of cool-down. Exercises gradually increased in difficulty and training load over the 16-week training period.

### 2.5. Statistical Analyses

Statistical analyses were performed using SPSS 17.0 and Microsoft Excel 2003 software. Data are expressed as mean ± SD. Changes in variables between pre- and posttraining and between groups were analyzed. A one-way analysis of variance (ANOVA) was used to examine the differences among the characteristics at baseline of the TC, PE, and control groups. Changes in the JPS, peak torque, and peak torque/weight between baseline and followup among the TC, PE, and control groups were compared using two-way (3 groups × 2 repetition assessments) repeated measures ANOVA. When ANOVA analysis revealed significant time and time-by-group interaction effects, paired *t*-tests were used to compare the changes in measures within groups. Statistical significance was assumed at *P* less than 0.05. 

We used five-point Likert scales to contrast the differences between the TC and PE groups with regard to health satisfaction and recommending others to participate in the project after 16 weeks of intervention [21]. With five-point scales, the points could be labeled as (1) 1: very satisfied; 2: satisfied; 3: neither satisfied nor dissatisfied; 4: dissatisfied; 5: very dissatisfied for health satisfaction; and (2) 1: strongly agree; 2: somewhat agree; 3: neutral/no opinion; 4: somewhat disagree; 5: strongly disagree about recommending that other people participate in the program. The Mann-Whitney Test was used to compare the differences between groups. Statistical significance was assumed at *P* less than 0.05.

## 3. Results

**Figure 1:**
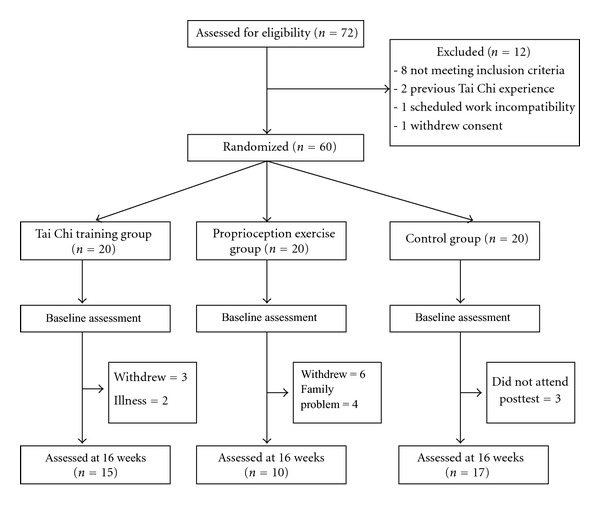
Flow diagram of eligibility assessment, exclusion, inclusion, and analysis.

The study design is outlined in Figure 1. Twelve of the 72 individuals initially recruited were deemed ineligible for study participation; eight subjects did not meet the inclusion criteria, two subjects had previous Tai Chi experience, one subject's work schedule was incompatible, and one subject withdrew consent. A total of 42 subjects completed the 16-week study program, and 18 subjects (TC *n* = 5, PE *n* = 10, control *n* = 3) were lost to followup. Reasons for dropout included illness, withdrawal, serious family problems, and not attending the final evaluation session. Hence, follow-up data were available for 15 of the 20 subjects in the TC group, 10 of the 20 subjects in the PE group, and 17 of the 20 subjects in the control group.  Table 1 lists baseline characteristics of the three groups. The groups were well matched at the baseline assessment, with no differences in key outcome variables apparent.

### 3.1. Ankle Joint Position Sense

**Figure 2:**
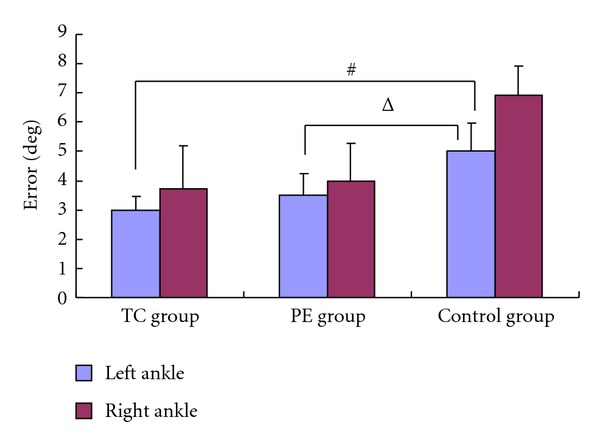
Joint position sense for 3 groups. ^#^There were significant differences between TC group and control group, *P* = 0.011.  ^Δ^There were significant differences between PE group and control group, *P* = 0.045. TC: Tai Chi; PE: proprioception exercise.

For the absolute error after the 16-week program (see Figure 2), (1) subjects in the TC group and PE group could be matching significantly more accurate than those in the control group (*P* = 0.014 for TC, *P* = 0.039 for PE) in the left ankle, (2) subjects in the TC group and PE group had a smaller amount of absolute error in the right ankle than the control group, but there were no significant differences (*P* = 0.184 for TC, *P* = 0.883 for PE), and (3) there were no significant differences in joint position matching of both ankles for the TC group and PE group (*P* = 0.979 for left, *P* = 0.184 for right). 

### 3.2. Muscle Strength

We found no significant differences in the strength and endurance of the bilateral ankle dorsiflexors and plantar flexors at a speed of 30°/s for concentric test conditions among groups (Table 2). 

### 3.3. Satisfaction Survey

**Figure 3:**
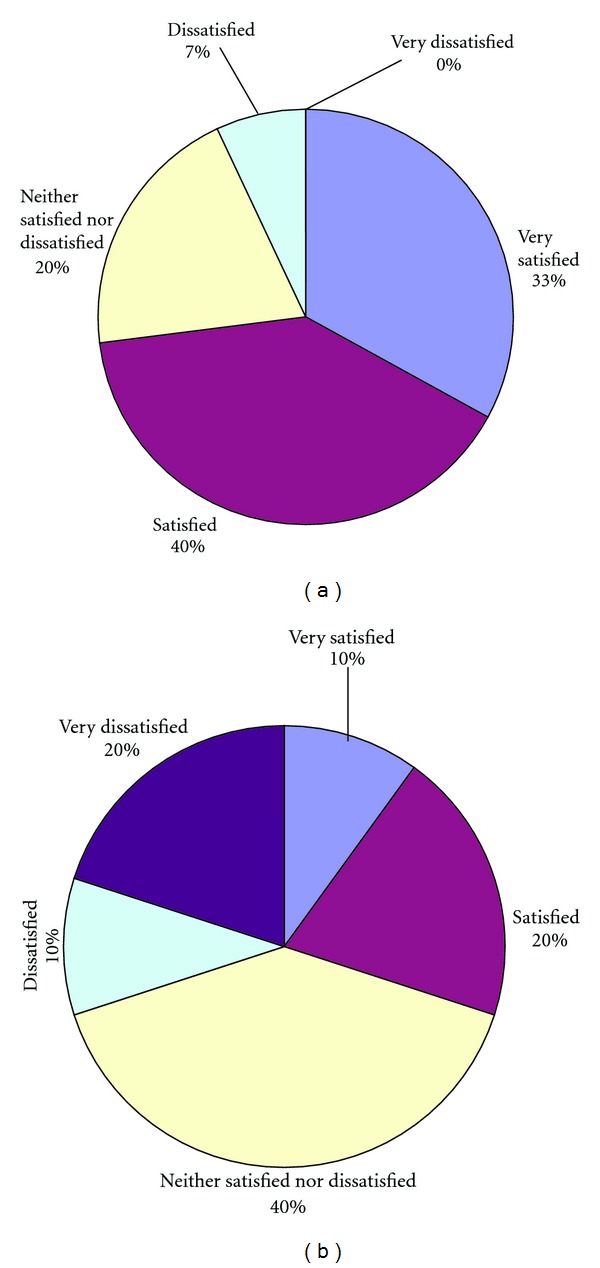
(a) Health satisfaction for Tai Chi group. (b) Health satisfaction for proprioception exercise group. The Mann-Whitney Test was used to compare the differences between groups. There were significant differences between PE group and control group, *P* = 0.036.

**Figure 4:**
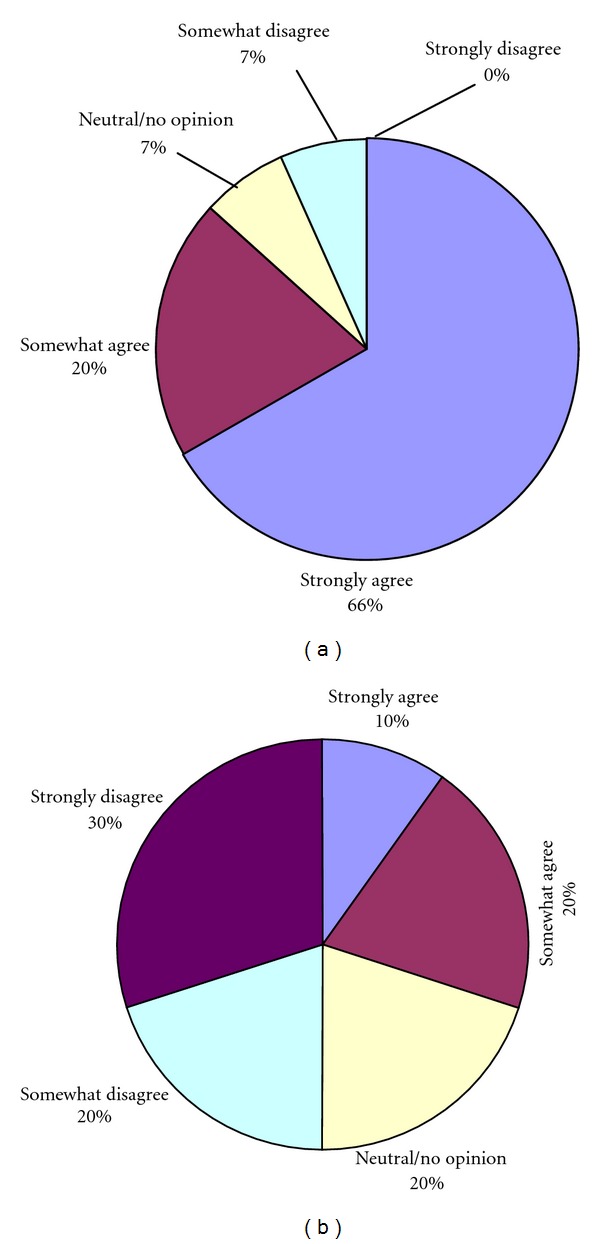
(a) Recommending other people learn the program for tai chi group. (b) Recommending other people learn the program for proprioception exercise group. The Mann-Whitney Test was used to compare the differences between groups. There were significant differences between PE group and control group, *P* = 0.002.

Elderly subjects expressed significantly higher health satisfaction in the TC group than in the PE group after 16 weeks (*P* = 0.036), and more subjects in the TC group recommended the program than did those in the PE group (Figures 3 and 4).

## 4. Discussion

### 4.1. Joint Position Sense

The TC and PE interventions evoked improvements in the joint position sense of bilateral ankles in this study. However, we were unable to show a difference after a 16-week exercise intervention between the TC group and PE group on a joint position sense. Leung et al. [22] performed a systematic and meta-analytical review of the effects of TC on balance function in elderly people. The systematic review based on 13 randomized controlled trials (RCTs) indicated that TC was effective in improving the balance function of elderly people. However, a TC program may not necessarily be superior to other interventions. Lelard et al. [10] assessed the effects of a TC program versus a balance training program on postural control and walking ability in elderly people. The authors measured static postural control and walking speed. They also did not find any significant modifications of postural parameters and walking speed between TC and balance training. Indeed, studies under more challenging postural conditions should be performed to verify this specific effect of TC training on proprioception function of the ankle in the daily living activities of older adults.

In addition, we found that the joint position sense of the left ankle was better than the right ankle in baseline values for the three groups. Significant matching of subjects in the TC group and PE group could be achieved more accurately than those in the control group (*P* = 0.014 for TC, *P* = 0.039 for PE) in the left ankle after 16 weeks of exercise. The result was similar to those reported previously in several studies [23–25]. Goble and Brown [23] found that for the proprioceptive matching task, errors were smaller for the nonpreferred left arm, whereas during the visual matching task, smaller errors were found for the preferred right arm. These results suggest a left-arm/right-hemisphere advantage for proprioceptive feedback processing and a right-arm/left-hemisphere advantage for visual information processing. Such asymmetries may reflect fundamental differences between the two-arm/hemisphere systems during the performance of bimanual tasks in which the preferred arm requires visual guidance to manipulate an object, whereas the nonpreferred limb stabilizes that object on the basis of proprioceptive feedback. The result of our study also showed that joint position sense of the left ankle was more sensitive to TC training and to the PE program than the right ankle.

### 4.2. Muscle Strength

Many researchers reported only mean peak torque values rather than values normalized by body weight. Normalizing by body weight is, thus, an important consideration for better comparison among subjects of varied body types. Additionally, as ankle sprains most usually occur in the closed kinetic chain, body weight also has an influence on the sprain moment generated at the ankle. Therefore, we consider peak torque/body weight a more relevant value compared with peak torque.

Our results affirm that subjects in the TC and PE groups did not have a larger amount of peak torque and peak torque/weight in both ankles than the control group, and there were no significant differences between groups. Perhaps, in order to improve muscle strength of the ankle joint, a 16-week TC intervention might not last long enough. For the lower limb muscle test, our results were consistent with previous studies [26–28], which showed that muscle strength of a TC program group for training lasting less than a year was not significantly higher than that of the control group. Wolf et al. [29] found that TC and elderly balance training participants with four months of training had no differences in ankle muscle strength. Another study confirmed that long-term TC exercisers with more than four years of experience showed significantly better muscle strength of the ankle joint compared with long-term regular joggers/swimmers and sedentary elderly people [30]. The results of these papers suggest that improving biomechanical characteristics of lower-extremity muscles may require a longer-lasting TC intervention for elderly people.

### 4.3. Satisfaction Survey

We did not find that the PE program has an improved effect than the TC program on proprioceptive function or muscle strength. However, the TC and PE groups showed a significant difference in health satisfaction and recommendation of the treatment to others after 16 weeks of practice. There may be several reasons for this result. First, a TC program is distinctive because it includes a structured cognitive component, also referred to as meditation [31]. In Tai Chi, a practitioner is required to choreograph slow movements according to visual imagery. In short, the mind directs the body in performing these movements. This makes TC different from other types of balance exercise. 

Brown et al. [32] provided the hypothesis that exercise plus cognitive strategy training programs are more effective than exercise programs lacking a structured cognitive component in promoting psychological benefits. But in proprioception exercise without a structured cognitive component, the practitioner's cognitive processing is random and unstructured. And Silsupadol et al. [33] suggested that cognitive dual-task training programs (balance exercise + cognitive training) were superior to single-task training (balance exercise) in improving balance function. Second, a PE program is not usually sufficiently interesting that the majority of elderly participants will maintain a long-term and regular habit of exercise. Lastly, subjects felt comfortable with the intensity of the program, and none reported discomfort while practicing Tai Chi. Participants were enthusiastic and made every effort to attend sessions. Our TC program proved to be effective, interesting, and convenient as a form of physical activity for older adults.

### 4.4. Limitations

Although our study elicited important observations regarding the usefulness of TC on the ankle in elderly people, there are some limitations to our research. First, because subjects had not learned the movements of tai chi previously, it sometimes proved difficult for subjects to correctly perform this exercise. We note that the present findings cannot be generalized to elderly people living in nursing homes or hospital settings, as these individuals are more likely to have limited mobility and/or a preestablished exercise program that does not permit physical interventions such as those assessed here. Finally, the sample size was too small to draw any firm conclusions. Further rigorous, multi-center RCTs with a large sample size are warranted.

## 5. Conclusion

Results of our study demonstrated similar effects of 16-week TC and proprioception exercise programs on joint position sense or muscle strength of ankle joint in elderly people. None of the outcome measures showed significant change posttraining in the TC or proprioception exercise groups. However, the elderly felt significantly more interested in TC program than PE program and also significantly more satisfactory to their health in the TC group than the PE group. Further study with long-term followup is needed to substantiate the role of Tai Chi exercise in the physical and psychological benefits. 

## Figures and Tables

**Table 1:** Baseline values for the 3 groups.

Variable	TC (*n* = 15)	PE (*n* = 10)	Control group (*n* = 17)	*P*
Men/female	7/8	4/6	7/10	0.932*
Age (y)	68.0 ± 1.41	68.8 ± 1.03	69.8 ± 0.73	0.663^#^
Body weight (kg)	60.73 ± 2.14	55.60 ± 2.73	59.59 ± 1.45	0.252^#^
Height (cm)	164.33 ± 2.09	167.30 ± 1.51	165.28 ± 0.95	0.528^#^

JPS test (°)				
Left ankle	4.20 ± 0.75	6.13 ± 1.77	4.16 ± 0.72	0.431^#^
Right ankle	8.93 ± 1.62	7.40 ± 1.43	8.77 ± 1.28	0.785^#^
PT (Nm): concentric, 30°/s				
Left plantar flexion	49.88 ± 6.81	46.68 ± 6.97	49.62 ± 4.59	0.934^#^
Right plantar flexion	49.08 ± 7.13	54.64 ± 8.03	55.55 ± 4.86	0.729^#^
Left dorsiflexion	14.4 ± 1.32	14.98 ± 2.36	14.60 ± 1.41	0.975^#^
Right dorsiflexion	14.75 ± 1.45	17.10 ± 2.63	16.95 ± 1.72	0.630^#^
PT/weight (Nm/kg): concentric, 30°/s				
Left plantar flexion	0.82 ± 0.09	0.85 ± 0.13	0.84 ± 0.082	0.978^#^
Right plantar flexion	0.82 ± 0.11	0.99 ± 0.14	0.95 ± 0.91	0.534^#^
Left dorsiflexion	0.24 ± 0.02	0.27 ± 0.04	0.24 ± 0.02	0.782^#^
Right dorsiflexion	0.25 ± 0.02	0.31 ± 0.04	0.29 ± 0.03	0.467^#^

Data reported as mean ± SD.

*Chi-square test.

^#^One-way analysis of variance.

TC: tai chi; PE: proprioception exercise; JPS: joint position sense; PT: peak torque.

**Table 2:** Muscle strength for the 3 groups.

Variable	TC	PE	Control group	P*
JPS test (°)				
Left ankle	3.0 ± 0.48^#^	3.5 ± 0.72^△^	5.03 ± 0.95	0.026
Right ankle	3.7 ± 1.49	4.0 ± 1.29	6.91 ± 0.99	0.083
PT (Nm): concentric, 30/°s				
Left plantar flexion	59.16 ± 6.71	61.4 ± 6.09	47.76 ± 3.69	0.138
Right plantar flexion	61.52 ± 7.72	69.53 ± 9.25	52.73 ± 4.37	0.221
Left dorsiflexion	17.84 ± 2.04	16.93 ± 2.71	13.11 ± 6.15	0.128
Right dorsiflexion	14.87 ± 1.37	18.02 ± 3.31	14.88 ± 1.37	0.079
PT/weight (Nm/kg): concentric, 30/°s				
Left plantar flexion	0.98 ± 0.1	1.13 ± 0.1	0.82 ± 0.04	0.11
Right plantar flexion	1.01 ± 0.1	1.25 ± 0.1	0.92 ± 0.1	0.19
Left dorsiflexion	0.31 ± 0.04	0.31 ± 0.05	0.22 ± 0.03	0.197
Right dorsiflexion	0.34 ± 0.03	0.32 ± 0.05	0.26 ± 0.03	0.150

Data reported as mean ± SD.

*A 2-way analysis of variance (group × time). ^#^There was significant differences between TC group and control group*P* = 0.011. ^△^There was significant differences between PE group and control group*P* = 0.045.

TC: tai chi; PE: proprioception exercise; PT: peak torque.
